# A pilot, randomized, placebo-controlled study of mindfulness meditation in treating insomnia in multiple sclerosis

**DOI:** 10.1186/s12883-023-03309-0

**Published:** 2023-07-11

**Authors:** Joseph B. Guarnaccia, Valentine Y. Njike, Anne Dutton, Rockiy G. Ayettey, Judith A. Treu, Beth P. Comerford, Rajita Sinha

**Affiliations:** 1 Multiple Sclerosis Treatment Center, Griffin Hospital, 350 Seymour Ave., Suite 1C, Derby, Connecticut 06418 USA; 2 Yale-Griffin Prevention Research Center, Griffin Hospital, 130 Division St., Derby, Connecticut 06418, USA; 3 Department of Psychiatry, Yale Stress Center, Yale University, New Haven, Connecticut 06510 USA

**Keywords:** Mindfulness, Multiple Sclerosis, Sleep Quality, Insomnia, Fatigue, Sleep Hygiene

## Abstract

**Objective:**

Mindfulness is an established approach to reduce distress and stress reactivity by improving awareness and tolerability of thoughts and emotions. This study compares mindfulness training to sleep hygiene in persons with multiple sclerosis (PWMS) who report chronic insomnia, examining sleep efficiency (SE), self-reported sleep quality and quality of life.

**Methods:**

Fifty-three PWMS were randomized (1:1) in a single-blinded, parallel group design to ten, two-hour weekly sessions of Mindfulness Based Stress Intervention for Insomnia (MBSI-I) over a span of ten weeks or a single, one hour sleep hygiene (SH) session over one day. The primary outcome measure was SE, measured by the Fitbit™ Charge 2 wrist device, at 10 and 16 weeks from the start of study interventions. Self-report outcomes included the Pittsburg Sleep Quality Rating Scale (PSQI), Insomnia Severity Index (ISI) and the Multiple Sclerosis Quality of Life Inventory (MSQLI). Nineteen participants in the MBSI-I group and 24 in the SH group completed the primary study. Subsequently, ten participants in the original SH group participated in the 10-week MSBI-I course and their data was added to the MBSI-I cohort (eMSBI-I).

**Results:**

While neither SE nor the PSQI showed significant differences between MBSI-I, eMBSI-I and SH groups, ISI improved in both the MSBI-I and eMBSI-I vs SH at 10 weeks (*p* = 0.0014 and *p* = 0.0275) but not 16 weeks. However, pre and post assessments within the MBSI-I and eMBSI-I cohorts did show significant improvement in the PSQI and ISI at 10 and 16 weeks, while SH was significant in the ISI only at 16 weeks. Several quality of life measurements, including fatigue, mental health and cognitive function favored the mindfulness cohorts.

**Conclusion:**

This pilot study demonstrates beneficial effects of MBSR on insomnia, sleep quality and quality of life in PWMS.

**Trial registration:**

NCT03949296. 14 May 2019.

## Introduction

Twenty to fifty percent of persons with multiple sclerosis (PWMS) report having chronic insomnia (CI) [1–4]. The causes can vary from neuropathic pain and paresthesias, muscle spasms, nocturia, obstructive sleep apnea, sleep related breathing disorders (SRBD), narcolepsy or restless legs syndrome, circadian rhythm disturbance to disease modifying treatments [1–9]. Primary insomnia in MS may result from central nervous system inflammation, involving activated microglia and astrocytes, inflammatory cytokines, oxidative stress, and dysregulation of adrenal cortical pathways, neurotransmission or sleep hormone cycles [10]. While involvement of specific anatomic regions with white matter plaques cannot be related to a primary insomnia pathway in MS, plaques located in specific regions of the cortical and subcortical regions of the brain, the brainstem or spinal cord can be related to insomnia via symptoms noted above [11, 12]. Studies showing that PWMS have a greater incidence of circadian rhythm disorders or abnormal melatonin secretion are inconclusive [13–17].

The clinical impact of chronic insomnia in PWMS, while frequently overlooked by clinicians, is supported by several studies demonstrating an overall lower quality of life [1, 2] and greater incidence of anxiety, depression and daytime fatigue [6, 8, 17]. Two hundred and six participants with MS and CI scored significantly higher on the Hospital Anxiety and Depression Scale, which measures anxiety and depression [1]. Similarly, PWMS who characterized themselves as poor vs. good sleepers scored significantly higher on the Multiple Sclerosis Impact Scale-29 anxiety and depression subscales [7]. Highlighting the association of depression and insomnia in MS, another study showed that non-depressed PWMS had a relatively low incidence of CI of 12.5% [18]. Insomnia also appears to be a risk factor for cognitive dysfunction in MS. A prospective, cross sectional study of PWMS showed that measures of poor sleep, including reduced sleep efficiency, increased nocturnal wakefulness and reduced REM sleep were correlated with worse performance in tests of global cognition, memory and attention [19].

Insomnia is often treated pharmacologically with antidepressants, anxiolytics, antihistamines, and benzodiazepines. Some PWMS self-medicate with cannabis [20]. However, these medications frequently have unacceptable side effects and certain risks, both psychological and physiological, including depression, cognitive dysfunction, daytime sedation, tolerance and dependence [21–23]. A potentially life-threatening condition arising combination of benzodiazepines and opioids is respiratory depression [21, 22].

Clearly, effective non-pharmacological treatments need to be explored to avoid these hazards. Two such programs, Mindfulness Based Stress Reduction (MBSR) and the Sleep Hygiene (SH) index have been used to treat CI. MBSR has its origins in non-Western Buddhist philosophy, and was developed by John Cabot Zin, PhD for stress reduction and anxiety [24]. MBSR is a program for managing stress that has been shown among other benefits, to improve CI, anxiety and depression [25–30].With respect to MS, two MBSR literature reviews showed significant improvements in quality of life, mental health and some physical health measures, including fatigue, standing, balance and pain [31, 32]. Another MS study showed that two different mindfulness techniques, each resulted in improved sleep as well as reduced anxiety, depression and fatigue [33]. By contrast, SH is a behavioral approach targeting environmental factors that interfere with sleep by means of recommendations based on a self-guided questionnaire [3, 34]. While requiring less commitment than MBSR, it effectiveness as a therapy for insomnia is not clear [34–40]. SH has not been studied in MS.

The purpose of this pilot study is to contrast the effectiveness of two therapies to treat CI in PWMS and their impact on subjective and objective measures of sleep, quality of life and actigraphy using the Fitbit™ Charge 2 band. We hypothesize that MBSI-I is superior to SH in improving sleep efficiency (SE) in PWMS with CI, and this will be associated with significant benefits in self-reported quality of life outcome measures compared to SH.

## Methods

### Study design

This randomized parallel, single-blinded clinical study enrolled 53 participants with MS who were randomly assigned (1:1) to attend ten, two-hour weekly sessions of MBSI-I or a one-hour counseling session on SH. Repeated assessments were performed at baseline, 10 and 16 weeks. The evaluator was blinded to treatment group assignment. The study was conducted at Griffin Hospital, a community hospital in the lower Naugatuck valley, in central CT, USA, in collaboration with the Yale Stress Center at the Yale School of Medicine in New Haven, CT. The study was approved by the Griffin Hospital Institutional Review Board (IRB) and registered on clinicaltrials.gov (NCT03949296) before initiating recruitment. The recruitment period commenced from May, 2019 to September, 2019.

Participants were assigned to one of two cohorts: one comprised of small groups of six to 11 persons who attended ten weekly sessions of MBSI-I (Mindfulness Based Stress Intervention for Insomnia) and the other, similar groups of participants who attended one sleep hygiene session conducted by the Griffin Hospital Sleep Wellness Center staff. MBSI-I is an adaptation of MBSR. Eighty percent attendance at the MBSI-I program was considered good compliance. Because of the lower than expected number of participants at the end of the randomized portion of the study, participants from the sleep hygiene cohort were offered participation in the MBSI-I course and repeated 10 and 16 week assessments after the course, i.e., the expanded MBSI-I, or eMBSI-I cohort. This was done to increase the statistical power of the results.

### Treatment groups

#### MBSI-I

In MBSR, participantsare taught under supervision to concentrate on the present moment intentionally and without judgment in order to reduce distress and emotional reactivity [24]. By becoming more aware of negative sensations, practitioners increase tolerance for negative thoughts and emotions. MBSR is supported by the neuroscience of stress and resilient adaptive behaviors. MBSI-I utilizes similar principles to teach a skill set using mindfulness, yoga and self-control to improve sleep. Techniques include therapeutic breath and synchronized yogic movement, focusing on the lower abdomen, along with instruction on cognitive and behavioral strategies to build self-control and promote healthy decision-making. A description of the MBSI-I sessions follows: (1) an orientation to introduce the concept of mindfulness, describe its potential benefits, and preview the content and logistics of subsequent sessions; (2) discussion and introduction of simple mindful practices and simple yoga poses within the context of mind–body medicine; (3) self-awareness of the role of perception and conditioning as a reaction to stressors and the integration of mindfulness into daily life; (4) exploration of challenges and insights encountered by participants as they practiced mindfulness on a daily basis; (5) the use of mindfulness to recognize and reduce negative, habitual stress reactivity with the development of more effective responses; (6) insights around reacting vs. responding to stressors, resulting in the use of mindfulness to make more objective and informed choices; (7) an emphasis on an emerging capacity to self-regulate and better cope with stressors and interpersonal communications challenges; (8) the practice of seamless continuity of moment-to-moment awareness through different mindfulness methods; (9) the cultivation of greater personal latitude and individuation of mindfulness practices, and (10) a review of participants’ overall experience with the program, and guidance in sustaining the momentum to continue mindfulness practices. The classes ranged in duration from one hour (#1) to two hours (# 2–7, 9- 10) and four hours for session #8. The MBSI-I class was taught by a qualified instructor from the Yale Stress Center (Anne Dutton) who had received training and certification from the Center for Mindfulness in Medicine, Health Care, and Society at the University of Massachusetts Medical School.

#### SH

This group attended a one-hour group counseling session based on a handout enumerating 15 sleep hygiene tips, published by the Centre for Clinical Intervention in Australia. The SH tips were as follows: (1) maintaining a consistent sleep pattern of going to bed and arising at about the same time each day; (2) attempting sleep only when feeling tired or sleepy; (3) getting up to do something calm until feeling sleepy and returning to bed if unable to sleep; (4) avoiding caffeine and nicotine for at least four to six hours before going to bed; (5) avoiding alcohol for at least four to six hours before going to bed; (6) using the bed only for sleeping and sex, which would preclude, among other activities, reading, watching television, or using a laptop; (7) avoiding naps during the day, or limiting them to less than an hour prior to 3 p.m.; (8) developing personalized rituals to relax and prepare for sleep; (9) taking a warm bath one to two hours before bedtime; (10) avoiding the tendency to check the clock frequently during the night; (11) using a sleep diary for a few weeks to track progress; (12) avoiding strenuous exercise within four hours of bedtime; (13) avoiding heavy meals before bedtime, and, if hungry, restricting oneself to a light snack; (14) creating a sleep environment that is quiet, comfortable, and dark, and (15) conducting a regular daytime routine, even after a night of poor sleep.

### Recruitment procedures and participants

Participants were recruited widely throughout the state of Connecticut via press releases distributed via paper and email to newspapers for articles and advertisements, MS support groups, neurologists, the Yale-Griffin Prevention Research Center electronic Newsflash, health magazines, and current and previous patients of the MS Treatment Center at Griffin Hospital (MSTC). Interested participants underwent an initial telephone screening to determine eligibility.

Inclusion criteria included a diagnosis of MS, based on the 2014 revised McDonald diagnostic criteria [41], age of at least 18 years, and moderate to severe insomnia based on the Insomnia Severity Index (ISI) [42]. Potential participants were excluded if they were diagnosed with sleep apnea or were at high risk, based on the STOP-Bang questionnaire [43]. Other exclusions included body mass index > 39, narcolepsy or other sleep-related disorders, expanded disability status scores (EDSS) > 7.0, history of alcohol or substance abuse as determined by the Principal Investigator, and other significant medical conditions. Excluded were persons who within the previous 30 days of screening had significant changes in medications or suffered from an MS relapse requiring the use of oral or intravenous corticosteroids.

After preliminary eligibility was established, a clinical screening was scheduled to determine final eligibility. These procedures included vital signs measurements, using calibrated equipment, of height, weight, waist circumference and blood pressure. A neurological exam and a brief physical exam were performed by the PI. The medical assessment included a description of insomnia symptoms and history of the diagnosis and treatment for MS as well as current medications and other pertinent medical information. Participants were consented using an IRB-approved Consent Form and told they could discontinue participation at any time during the study without penalty.

### Randomization and blinding

The former was carried out using SAS software for Windows version 9.4 (SAS Institute, Cary, NC) by dividing participants into blocks of 14, 17, and 22. The study coordinator enrolled the participants and assigned them to one of the two treatment groups based on the randomization algorithm. Therefore, the coordinator was unblinded and aware of the randomization scheme. The Principal Investigator (PI), statistician and study personnel assessing outcome measures were blinded to the treatment assignments throughout the study. Participants were labelled as receiving either treatment A or B. Only the study coordinator knew the treatment allocations that each participant received. Participants’ group assignment was unmasked by the study coordinator at conclusion of statistical analyses.

### Outcome measures

#### Primary outcome

The study’s primary outcome was sleep quality defined by sleep efficiency, as measured by the Fitbit™ Charge 2 wrist device. This is a consumer wristband-tracking device that embeds a heart rate monitor and three-axis accelerometer to report heart rate, exercise and sleep. Raw data from the device was uploaded to Fitbit, which processed it using a proprietary algorithm. Data reported back from Fitbit included subject ID, date of sleep, start time, end time, minutes asleep, minutes in sleep period and sleep efficiency. Other parameters, were also reported, including sleep stages, but this data was not deemed reliable enough to use in the analysis. All statistics on sleep data were performed by our Study Statistician as noted below.

The FitBit™ Charge 2 was introduced in 2016 and replaced in 2019 by improved devices. Cost, ease of use by persons in a natural sleep environment, the amount of data collected, the inessential requirement for specialized technicians to interpret the date are some of the advantages of consumer actigraphy over the gold standard polysomnography (PSG) [44].

Sleep efficiency is defined as the percentage of time asleep while in bed during a specified sleep period. A normal sleep efficiency is at least 85%. This was calculated from the longest recorded sleep period (> = two hours) during a 24 h period that occurred within or overlapped between 8:00 pm and 8:00 am. In order to distinguish a sleep period from a daytime nap, the onset, but not the end of the sleep period, had to fall within the sleep window [45]. The Fitbit™ device was assigned to participants at the baseline visit and they were instructed to wear the device constantly for the duration of the study. i.e., from the beginning of the 10-week intervention to the end of the 16 week post-intervention period. They were further instructed on how to synchronize their device with their cell phone and computer and asked to upload their sleep data on a daily basis. For the purpose of assessing the impact of the intervention on objective sleep quality, the first week of Fitbit sleep data collected during the 10-week intervention (i.e., during the week of the mindfulness orientation session) counted as baseline data; the last week of the 10-week intervention counted as post-intervention data; and the last week of the 16-week post-intervention period was used to assess the sustainability of the intervention. Whenever possible seven sleep periods over seven consecutive days of sleep were averaged, starting on the day of the baseline and 10-week visits and the prior week ending on the day of the final 16-week visit. Adjustments were made when participants, for instance, encountered technical difficulties in setting up and/or syncing their Fitbit™, whose Fitbit™ data collection ended before the date of their final scheduled visit or who had recording gaps during the 7-day periods relative to their visits. In those cases, we used the closest 7-day periods relative to visit dates or seven nonconsecutive days and the prior week ending on the day of the final 16-week visit. Data was sent to Fitbit as a batch file at periodic intervals.

#### Secondary outcomes

These included self-reported sleep quality as measured by the Pittsburgh Sleep Quality Index (PSQI) at baseline, the end of the 10-week intervention, and 16-weeks post-intervention. The PSQI is a self-rated questionnaire to assess perceived sleep quality and disturbances over the prior one-month time interval [46, 47]. This 19-item instrument uses a Likert scale (ranging from 0 to 3) to assess 7 clinically derived domains of sleep: sleep quality, sleep latency, sleep duration, habitual sleep efficiency, sleep disturbances, use of sleeping medication, and daytime dysfunction, which are scored individually. The sum of scores for these seven components yields one global score. Clinical and clinometric properties of the PSQI were assessed over an 18-month period with "good" vs. "poor" sleepers. A global score > 5 yielded a diagnostic sensitivity of 89.6% and specificity of 86.5% (kappa = 0.75, *p*< 0.001) in distinguishing “good” vs. “poor” sleepers [47]. However, scores greater than 8.0 might be more sensitive to detect poor sleep quality in chronic disease populations.

The Insomnia Severity Index (ISI) is a brief self-report screening tool of seven questions assessing sleep over the previous two weeks, including three questions rating difficulties with: 1) falling asleep; 2) staying awake and 3) early morning awakening on a five-point Likert scale ( ‘0’ = none to ‘4’ = very severe). Other questions rate dissatisfaction with current sleep pattern (‘0’ = very satisfied to ‘4’ = very dissatisfied); how noticeable sleep problems are to others (‘0’ = not at all to ‘4’ = very much); worried/distressed about current sleep problem (‘0’ = not at all to ‘4’ = very much) and interference with daily function (‘0’ = not at all to ‘4’ = very much). Total scores of 15 to 21 indicate moderate and scores of 22–28 indicate severe insomnia (www.myhealth.va.gov). The ISI shows good internal consistency and significant correlation with sleep diaries and polysomnography [42].

Other secondary outcome measures included the self-reported Multiple Sclerosis Quality of Life Inventory (MSQLI) [48, 49]. The MSQLI includes a set of 10 questionnaires to provide a quality of life measure that is both generic and MS-specific.. These scales include the Short Form 36 health survey questionnaire (SF-36), Modified Fatigue Impact Scale (MFIS), Medical Outcomes Surgery (MOS) Pain Effects Scale, Sexual Satisfaction Scale, Bladder and Bowel Control Scales, Visual Impairment Scale, Self-Reported Cognitive Dysfunction scale (SRCD), Mental Health Inventory (MHI), Modified MOS Social Support Survey Score, and Kurtske Expanded Disability Status Scale (EDSS). Each individual scale generates a separate score. There is no global composite combining all the scales into a single score. There is good internal consistency reliability for the subscales of the MSQLI, with the lowest alpha being 0.67 (for social functioning on SF-36). Other coefficients range from 0.78 (BWCS) to 0.97 (MSSS). Test–retest reliability on the SF-36 ranges from 0.60 (social functioning) to 0.81 (physical functioning) [48].

Muscle spasticity was measured by the Modified Ashworth Scale (MAS) [50], and self-reported restless leg syndrome severity by the International Restless Legs Scale (IRLS) [51]. Data on medication changes or supplement use were also collected.

The Expanded Disability Status Scale (EDSS) is a standard measure of physical and mental impairment in MS that is universally employed in MS studies and clinical practice. It consists of a set of subscale, measuring different neurological functions, and a ten-point ordinal scale that grades neurological findings in MS, ranging from no impairment (0), moderate disability (3.0 or higher), reliance on a unilateral assistive device to walk 100 m (6.0), wheelchair bound (7.0) and death from MS (10) (reference). The Principal Investigator, a Board Certified neurologist with more than two decades of experience in treating MS and participating in MS clinical trials, performed the EDSS examinations.

#### Exploratory outcome measures

Within-group comparisons comparing baseline to 10 weeks and 16 weeks were done for the MBSI-I and SH cohorts. At the end of the randomized phase, participants in the sleep hygiene group were offered the same MBSI-I training and analyzed as a group, the expanded MBSI-I cohort (eMBSI-I). The eMBSI-I outcomes analyses included data from the original MBSI-I cohort as well as the crossover SH participants. Within-group and between group (SH) analyses were performed for the eMBSI-I cohort at the same time points.

### Adverse events reporting scheme

Adverse events, including MS relapses, were recorded throughout the study by the coordinator. These were presented to the PI, who would inform the IRB as per the protocol.

### Statistical analysis

The sample size estimate allowed for 20% attrition and noncompliance to provide ≥ 80% power and maximum type I error of 5% to detect a minimal difference of 1.6 point improvement in subjective sleep quality as measured by the ISI sleep scale between cohorts. Generalized linear models were used to compare scores of the outcome measures between cohorts. Paired student t-tests were used to assess difference from baseline to endpoints for each group. Regression models were used to control for covariates (i.e., age, gender, race, compliance, and medication use). All analyses at endpoints were based on intention-to-treat principle. SAS software for Windows version 9.4 (SAS Institute, Cary, NC) was used to carry out all statistical analyses. *P*-values of < 0.05 were considered statistically significant. Data are presented as mean ± standard deviation except otherwise stated. Positive changes from baseline indicate improvement in the Quality of Life, mental health inventory, and social support scales. Negative changes from baseline indicate improvement in the other measures (i.e., Fitbit, PSQI, Modified Fatigue Impact Scale, MOS Pain Effects Scale, Sexual Satisfaction Scale, Bladder Control Scale, Bowel Control Scale; Impact of Visual Impairment Scale, Self-Reported Cognitive Dysfunction, ISI, EDSS, MAS, and IRLS).

### Role of the funding sources

Neither Fitbit, Inc., which provided the Fitbit™ Charge 2 device as well as data tabulation free of charge, nor the funder of the study, had any role in study design, data collection, data analysis, data interpretation, or writing of the report.

### Compliance

Our methods followed the Consort-2010 reporting guidelines (Schulz KF, Altman DG, Moher D, for the CONSORT Group. CONSORT 2010 Statement: updated guidelines for reporting parallel group randomized trials).

## Results

### Study participants

**Fig. 1:**
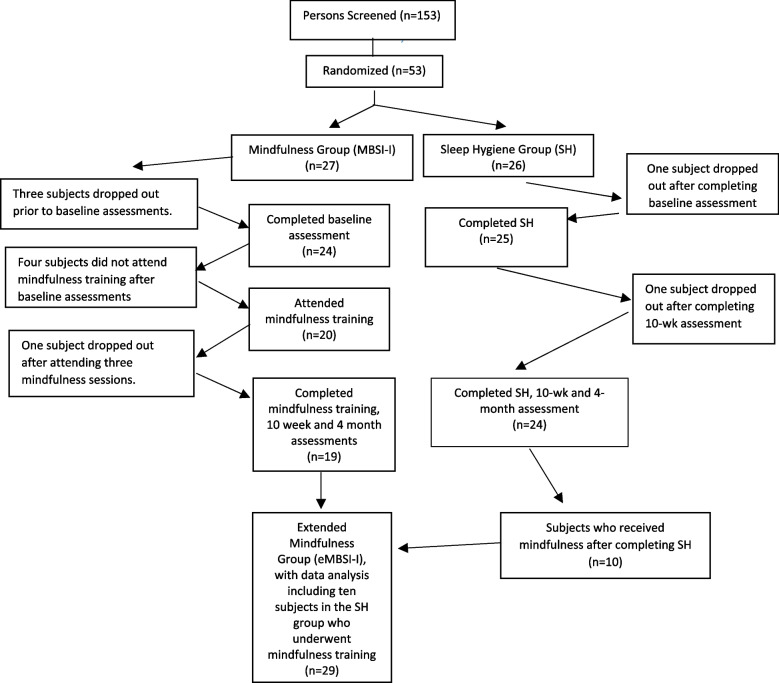
Study Consort diagram

Of 153 potential participants screened, 53 were enrolled and randomized to MBSI-I or SH (see CONSORT Fig. 1). Eight persons dropped out after randomization to the MBSI-I cohort. Six persons seemed to lose interest and did not respond to follow-up messages prior to the start of the intervention. One person was unable to continue after completing three MBSI-I sessions due to other commitments. Another was not compliant with the intervention and did not respond to the study team. Two participants in the SH group dropped out, one after completing baseline assessments and the other after completing the 10-week assessment. Forty-three participants completed the study. The cohorts were predominantly female with an average age of 51 years. The MBSI-I and SH cohorts were comparable in age, gender, body mass index and blood pressure, as well as baseline values of primary and secondary measures, including Fitbit™ data (Tables 1 and 2). Of the seven subjects who dropped out of the study after completing baseline assessments, there were no demographic or baselines differences between the dropouts and the completed subjects. The baseline study population met criteria for moderately severe insomnia with scores of 9.5 to 9.8 on the PSQI and 16 on the ISI. The total MSFIS score range from 46 to 49 is above the cutoff of 38 for persons with fatigue.

**Table 1:** Baseline demographic and study variables: MBSI-I (mindfulness based sleep intervention for insomnia) vs. SH (sleep hygiene) groups (*p* values nonsignificant)

**Variable**	**MBSI-I (24**)	**SH (26**)
Gender		
Male	4 (8.0%)	5 (10.0%)
Female	20 (40.0%)	21 (42.0%)
Age (years)	50.8 ± 10.1	50.9 ± 10.8
BMI (kg/m^2^)	26.7 ± 3.5	28.1 ± 5.1
Systolic Blood Pressure (mmHg)	118.9 ± 12.2	121.0 ± 12.8
Diastolic Blood Pressure (mmHg)	73.1 ± 9.8	74.2 ± 9.6
**Quality of Life**		
Physical Function Scale	57.8 ± 29.4	53.2 ± 29.1
Role-Physical Scale	25.0 ± 33.7	28.3 ± 36.4
Bodily Pain Scale	50.1 ± 28.1	41.0 ± 20.0
General Health Scale	48.8 ± 15.8	46.9 ± 12.8
Vitality Scale	27.8 ± 19.4	27.7 ± 23.8
Social Functioning Scale	56.5 ± 25.2	53.3 ± 30.9
Role-Emotional Scale	52.2 ± 38.7	54.2 ± 40.3
Mental Health Scale	58.9 ± 20.4	56.3 ± 23.9
Physical Components Summary Scale	36.0 ± 11.9	33.5 ± 9.2
Mental Component Summary Scale	40.4 ± 10.9	39.1 ± 12.2
**Modified Fatigue Impact Scale**		
Physical Subscale	21.5 ± 7.2	23.2 ± 8.9
Cognitive Subscale	21.5 ± 8.5	21.5 ± 10.2
Psychosocial Subscale	4.1 ± 2.0	4.7 ± 2.3
Modified Fatigue Impact Scale Total Score	46.8 ± 16.3	49.2 ± 20.4
Modified fatigue 5 Items scale	11.0 ± 4.3	11.5 ± 5.2
MOS Pain Effects Scale	16.8 ± 5.7	18.4 ± 6.7
Sexual Satisfaction Scale	16.4 ± 6.8	14.2 ± 6.6
Bladder Control Scale	4.7 ± 4.6	7.2 ± 7.4
Bowel Control Scale	3.1 ± 3.9	5.4 ± 6.1
Impact of Visual Impairment Scale	2.0 ± 2.3	1.8 ± 2.1
**Self-Reported Cognitive Dysfunction**		
Attention/Concentration Subscale	10.1 ± 5.0	11.2 ± 4.9
Retrospective Memory Subscale	8.4 ± 4.6	9.2 ± 4.9
Prospective Memory Subscale	7.0 ± 3.6	8.0 ± 5.4
Planning/Organization Subscale	9.8 ± 4.4	9.8 ± 5.4
PDQ Total Score	35.3 ± 16.2	39.6 ± 19.6
PDQ 5 items Scale	8.8 ± 4.6	9.9 ± 5.1
**Mental Health Inventory**		
Anxiety Subscale	52.5 ± 22.8	53.2 ± 24.9
Depression Subscale	62.8 ± 19.6	60.8 ± 26.0
Behavior Control Subscale	70.9 ± 18.3	69.8 ± 23.1
Positive Affect Subscale	44.1 ± 19.9	46.9 ± 23.4
Mental Health Inventory Total Score	57.3 ± 18.1	57.8 ± 21.9
Modified MOS Social Support Survey Score	62.4 ± 23.6	65.4 ± 24.7
Expanded Disability Status Scale	2.7 ± 1.3	2.5 ± 1.5
Ashworth Scale score	1.3 ± 3.0	1.6 ± 3.1
Restless Legs Syndrome score	3.3 ± 7.4	5.9 ± 10.1
**Pittsburg Sleep Quality Index (PSQI)**		
**Global Score**	9.5 ± 2.7	9.8 ± 3.2
**Component Scores**		
Sleep duration	0.3 ± 0.6	0.3 ± 0.9
Sleep disturbance	1.9 ± 0.6	2.1 ± 0.5
Sleep latency	2.0 ± 1.0	2.0 ± 1.1
Daytime dysfunction	1.5 ± 0.6	1.4 ± 0.7
Habitual sleep efficiency	0.2 ± 0.5	0.3 ± 0.8
Sleep quality	2.0 ± 0.9	2.0 ± 0.7
Use of sleep meds	1.6 ± 1.3	1.8 ± 1.3
**Insomnia Severity Index Score**	16.7 ± 3.8	16.5 ± 5.9

**Table 2:** Fitbit™ data: baseline values

**Sleep Variables**	**Mindfulness (*n* = 20)**	**Sleep Hygiene (*n* = 23)**	**Pr >|t|**
Sleep time (mins)	409.1 ± 66.3	407.3 ± 60.0	0.9247
Time in bed (mins)	459.4 ± 79.6	463.7 ± 73.1	0.8563
Sleep efficiency (%)	89.4 ± 3.1	88.0 ± 3.8	0.2176

Overall compliance with the SH and MBSI-I classes met our prespecified goal. All subjects in the SH group (*n* = 24) attended the sleep hygiene session. The compliance rate for both the MBSI-I and eMBSI-I cohorts 83% for the ten sessions. In the MBSI-I group, three participants attended 10/10 classes, five attended 9/10 classes, eight attended 8/10 classes, one attended 7/10 classes and two attended 6/10 classes. In the crossover SH to eMBSI-I group, three attended 10/10 classes, two attended 9/10 classes, one attended 6/10 classes and one 5/10 classes.

### Primary outcome of sleep efficiency: MBSI-I vs. SH at 10 and 16 weeks

MBSI-I did not show superiority over SH in terms over sleep efficiency at 10 or 16 weeks. SE did not improve over the course of the study either within or between cohorts or when the eMBSI-I cohort was analyzed (Tables 3 and 4).

**Table 3:** Baseline to 10 weeks. Change in sleep parameters measured by Fitbit™ (Δ sleep time/Δ time in bed = Δ SE)

**Sleep parameters (abs change)**	**MBSI-I** **(20)**	**MBSI-I** **(w/i grp)** **Pr >|t|**	**SH** **(23)**	**SH** **(w/i grp)** **Pr >|t|**	**MBSI vs. SH** **Pr >|t|**	**eMBSI-I** **(25)**	**eMBSI-I (w/i grp)** **Pr >|t|**	**eMBSI-I vs. SH** **Pr >|t|**
sleep time (mins)	-23.0 ± 48.4	0.0596	-0.6 ± 47.1	0.9509	0.1527	-28.5 ± 54.7	0.0156	0.0741
time in bed (mins)	-31.4 ± 50.1	0.0167	2.4 ± 49.4	0.8290	0.0416	-32.7 ± 53.7	0.0056	0.0272
SE (%)	1.1 ± 3.3	0.1908	-0.5 ± 4.2	0.6251	0.2248	-0.0 ± 4.5	0.9982	0.7254

**Table 4:** Baseline to 16 weeks. Change in sleep parameters measured by Fitbit (Δ sleep time/Δ time in bed = Δ SE)

**Sleep parameters (abs change)**	**MBSI-I** **(20)**	**MBSI-I (w/i grp)** **Pr >|t|**	**SH** **(23)**	**SH** **(w/i grp)** **Pr >|t|**	**MBSI-I vs. SH** **Pr >|t|**	**eMBSI-I** **(24)**	**eMBSI-I (w/i grp)** **Pr >|t|**	**eMBSI-I vs. SH** **Pr >|t|**
sleep time (mins)	-10.3 ± 55.4	0.4562	4.3 ± 58.2	0.7352	0.4355	-10.0 ± 54.9	0.3801	0.1527
time in bed (mins)	-9.6 ± 60.5	0.5218	-1.3 ± 66.3	0.9264	0.6899	-10.9 ± 61.1	0.3926	0.0416
SE (%)	-0.5 ± 3.1	0.5039	1.1 ± 3.7	0.1673	0.1493	-0.3 ± 3.2	0.6824	0.2248

### Secondary sleep outcomes

The MBSI-I cohort and the eMBSI-I cohort spent significantly less time in bed at 10 weeks relative to baseline when compared to the SH cohort (MBSI-I vs SH: *p* < 0.0416; eMBSI-I vs SH: 0.0272). Within each cohort, time spent in bed was significantly reduced within the MBSI-I and the eMBSI-I cohorts at 10 weeks relative to baseline (MBSI-I: *p* < 0.0167; eMBSI-I: *p* < 0.0056) but not in the SH cohort (*p* < 0.82) (Table 3). This finding was extended to 16 weeks only in the eMBSI-I cohort relative to the SH group (eMBSI-I vs. SH: *p* < 0.0416) (Table 4).

### MBSI-I, eMBSI-I and SH interventions and self-reported sleep outcomes

The MBSI-I cohort did not show improvement relative to SH at 10 or 16 weeks in the self-reported Global PSQI. However, within group improvements in the Global PSQI scores for the MBSI-I cohorts relative to baseline were significant (10 weeks: MBSI-I: *p* < 0.0296; eMBSI: *p* < 0.0025; 16 weeks: MSBI-I: *p* < 0.0049; eMSBI-I: *p* < 0.0012). The Global PSQI was not significant for the SH cohort (10 weeks: *p* < 0.0.7496; 16 weeks: *p* < 0.0.6287) (Tables 5 and 6).

**Table 5:** Baseline to 10 weeks. Comparison between MBSI-I, eMBSI-I, SH on self-reported sleep measures

**Variable**	**MBSI-I** **(24)**	**MSBI-I (w/i grp)** ***P* >|t|**	**SH** **(25)**	**SH** **(w/i grp)** ***P* >|t|**	**MSBI-I vs. SH** ***P* >|t|**	**eMBSI-I (29)**	**eMBSI-I (w/i grp)** ***P* >|t|**	**eMBSI-I** **vs SH** ***P* >|t|**
**PSQI**								
**Global Score**	-1.3 ± 2.4	0.0296	-0.2 ± 2.5	0.7496	0.1296	-1.3 ± 2.1	0.0025	0.0718
**Component Scores**								
Sleep duration	0 ± 0.3	1.0000	-0.2 ± 0.9	0.3824	0.4162	0.1 ± 0.5	0.4238	0.2600
Sleep disturbance	0 ± 0.7	1.0000	-0.04 ± 0.5	0.7136	0.8268	-0.03 ± 0.6	0.7689	0.9727
Sleep latency	-0.4 ± 0.8	0.0281	-0.04 ± 1.0	0.8462	0.1811	-0.4 ± 0.9	0.0157	0.1513
Day dysfunction	-0.1 ± 0.8	0.5778	0.2 ± 0.8	0.1615	0.1747	-0.4 ± 0.9	0.0387	0.0140
Habitual sleep efficiency	-0.06 ± 0.7	0.7492	-0.2 ± 0.6	0.0961	0.4554	0.1 ± 0.7	0.6021	0.1342
Overall Sleep Quality	-0.6 ± 0.8	0.0039	-0.2 ± 0.7	0.1701	0.0785	-0.5 ± 0.7	0.0007	0.0995
Need Meds to Sleep	-0.1 ± 0.9	0.6301	0.2 ± 1.1	0.4450	0.3796	0.1 ± 0.8	0.6455	0.3537
**Insomnia Severity Index Score**	-5.6 ± 4.2	0.0001	-1.3 ± 3.9	0.1242	0.0014	-4.2 ± 5.0	0.0001	0.0275

**Table 6:** Baseline to 16 weeks. Comparison between MBSI-I, eMBSI-I, SH on self-reported sleep measures

**Variable**	**MBSI-I (24)**	**MSBI-I (w/i grp)** ***P* >|t|**	**SH** **(25)**	**SH** **(w/i grp)** ***P* >|t|**	**MBSI-I** **vs. SH** ***P* >|t|**	**eMBSI-I (29)**	**eMBSI-I** **(w/i grp.)** ***P* >|t|**	**eMBSI-I** **vs. SH** ***P* >|t|**
**PSQI**								
**Global Score**	-2.0 ± 1.8	0.0049	-0.5 ± 3.8	0.6287	0.2081	-1.7 ± 2.1	0.0012	0.2868
**Component Scores**								
Sleep duration	-0.2 ± 0.4	0.1669	-0.1 ± 1.1	0.8078	0.7566	-0.1 ± 0.3	0.1623	0.9547
Sleep disturbance	-0.2 ± 0.6	0.3409	-0.2 ± 0.7	0.2722	0.9038	-0.1 ± 0.6	0.4930	0.6020
Sleep latency	-0.5 ± 0.7	0.0251	0 ± 1.2	1.0000	0.1864	-0.4 ± 0.7	0.0160	0.1953
Day dysfunction	-0.4 ± 0.5	0.0379	0 ± 0.9	1.0000	0.2070	-0.7 ± 0.6	< .0001	0.0104
Habitual sleep efficiency	-0.1 ± 0.3	0.3434	0.1 ± 1.0	0.7938	0.5702	0.1 ± 0.5	0.3306	0.9275
Overall Sleep Quality	-0.8 ± 1.0	0.0200	-0.4 ± 0.9	0.0823	0.2992	-0.7 ± 0.8	0.0009	0.3358
Need Meds to Sleep	0.2 ± 1.3	0.6400	0.1 ± 1.1	0.6349	0.9347	0.2 ± 1.1	0.4462	0.9022
**Insomnia Severity Index Score**	-5.8 ± 3.6	0.0001	-3.9 ± 6.8	0.0127	0.2391	-3.8 ± 4.8	0.0002	0.9624

The robust effect of MBSI-I was observed for other sleep outcomes. The ISI was significantly improved at 10 weeks relative to baseline for the MBSI-I cohorts (MBSI-I vs. SH: *p* < 0.0014 and eMBSI-I vs. SH: *p* < 0.0275) (Table 5). However, the ISI improved significantly in the SH cohort relative to baseline at 16 weeks (*p* = 0.0127). While this improvement was not as robust as for the MBSI-I and eMBSI-I cohorts (*p* = 0.0001 and *p* = 0.0002 respectively), this eliminated the benefit shown for the mindfulness cohorts at 16 weeks.

Other component scores of the PSQL showed strong within-in group effects in the mindfulness cohorts. This was observed in sleep latency and overall sleep quality in both MBSI-I and eMBSI-I cohorts at 10-and 16-weeks (*p* < 0.05). At 10-weeks, daytime dysfunction due to sleepiness was improved relative to baseline within the eMBSI-I group (*p* < 0.0387) and in comparison to SH (*p* < 0.014) but not within the MBSI-I or the SH cohorts. On the other hand, at 16 weeks, day dysfunction due to sleepiness was improved relative to baseline within the MBSI-I (*p* < 0.0379) and the eMBSI-I (*p* < 0.0001) cohorts. Day dysfunction was not improved in the MBSI-I vs. SH groups at 10 or 16 weeks. However, day dysfunction was significantly improved for the eMSBI-I cohorts vs SH cohort at both 10 and 16 weeks (*p* < 0.0104 for both) (Tables 5 and 6).

### Secondary outcomes: MBSI-I, eMBSI-I and SH and self-reported quality of life

Relative to SH, the MBSI-I cohorts showed significant improvement at 10 weeks in bowel control (MBSI-I vs. SH: *p* < 0.0142), vitality scale (eMBSI-I vs SH: *p* < 0.0471) and the positive affect subscale of the Mental Health Inventory (eMBSI vs. SH: *p* < 0.0287) (Table 7). At 16 weeks, significant improvement was observed on the cognitive subscale of the MFIS (MBSI-I vs. SH: *p* < 0.0191and eMBSI-I vs. SH: *p* < 0.0018), the MFIS total score (MBSI-I vs. SH: *p* < 0.0411 and eMBSI-I vs. SH: *p* < 0.0051), the modified fatigue 5 items scale (MBSI-I vs. SH: *p* < 0.0655 and eMBSI-I vs. SH *p* < 0.0200) and the planning organizational subscale of the self-reported Cognitive Dysfunction scale (eMBSI-I vs SH: *p* < 0.0331) (Table 8).

**Table 7:** Baseline to 10 weeks. Comparisons between MBSI-I, eMBSI-I, and SH cohorts on quality of life variables

**Variable**	**MBSI-I** **(24)**	**MBSI-I** **(w/i grp.)** ***P* >|t|**	**SH (*n* = 25)**	**SH** **(w/i grp.)** ***P* >|t|**	**MBSI-I** **vs. SH** ***P* >|t|**	**eMBSI-I** **(29)**	**eMBSI-I** **(w/i grp.)** ***P* >|t|**	**eMBSI-I** **vs. SH** ***P* >|t|**
Quality of Life **+**								
Physical Function Scale	2.5 ± 13.2	0.4328	-3.3 ± 14.2	0.2939	0.1942	3.2 ± 11.3	0.1446	0.0786
Role-Physical Scale	4.2 ± 32.4	0.5921	-6.5 ± 29.4	0.2990	0.2759	0.9 ± 42.8	0.9128	0.4839
Bodily Pain Scale	0.8 ± 16.6	0.8452	-2.8 ± 14.2	0.3488	0.4583	0.4 ± 15.1	0.8915	0.4397
General Health Scale	0.4 ± 7.5	0.8037	0.6 ± 12.2	0.8161	0.9638	0.8 ± 7.6	0.6232	0.9638
Vitality Scale	3.9 ± 18.6	0.3873	-3.3 ± 14.9	0.2834	0.1694	5.4 ± 15.7	0.0830	0.0471
Social Functioning Scale	6.3 ± 24.0	0.2840	3.8 ± 24.3	0.4600	0.7492	5.8 ± 22.7	0.1871	0.7628
Role-Emotional Scale	3.7 ± 47.0	0.7421	-4.2 ± 39.7	0.6120	0.9726	-2.4 ± 46.2	0.7871	0.8828
Mental Health Scale	4.0 ± 13.4	0.2367	0.5 ± 14.8	0.8672	0.4493	6.1 ± 13.7	0.0294	0.1747
Physical Components Summary Scale	1.2 ± 7.3	0.5044	-1.2 ± 4.4	0.2792	0.2560	0.5 ± 6.6	0.6859	0.3510
Mental Component Summary Scale	1.5 ± 9.8	0.5469	1.8 ± 8.3	0.3804	0.9075	2.3 ± 9.8	0.2276	0.8600
Modified Fatigue Impact Scale-								
Physical Subscale	-1.2 ± 4.1	0.2587	0.3 ± 5.2	0.7823	0.3403	-1.8 ± 4.7	0.0468	0.1246
Cognitive Subscale	-1.4 ± 4.9	0.2281	-0.2 ± 8.2	0.9200	0.5423	-2.4 ± 5.0	0.0223	0.2632
Psychosocial Subscale	0.1 ± 1.8	0.7909	-0.3 ± 1.4	0.3277	0.4220	0.0 ± 1.8	1.0000	0.5214
Modified Fatigue Impact Scale Total Score	-2.2 ± 8.1	0.2862	0.4 ± 13.6	0.8889	0.4652	-4.1 ± 9.9	0.0392	0.1814
Modified fatigue 5 Items scale	-0.6 ± 2.5	0.3680	0.3 ± 3.6	0.6507	0.3738	-0.9 ± 2.7	0.0806	0.1532
MOS Pain Effects Scale-	0.7 ± 4.6	0.5469	0.3 ± 4.3	0.7771	0.7638	-0.9 ± 5.1	0.3794	0.4035
Sexual Satisfaction Scale-	-2.1 ± 3.7	0.0663	0.7 ± 4.3	0.5206	0.0744	-1.4 ± 4.3	0.1783	0.1590
Bladder Control Scale-	0.6 ± 4.0	0.5287	0.2 ± 4.5	0.8198	0.7734	-0.2 ± 3.6	0.7956	0.7295
Bowel Control Scale-	1.5 ± 2.0*	0.0062	-0.6 ± 3.0	0.3381	0.0142	0.8 ± 3.6	0.2782	0.1532
Impact of Visual Impairment Scale-	0.3 ± 1.6	0.4516	0.1 ± 1.2	0.7395	0.6305	-0.04 ± 1.5	0.9010	0.7590
Self-Reported Cognitive Dysfunction-								
Attention/Concentration Subscale	-0.3 ± 2.9	0.6902	-0.1 ± 3.5	0.9078	0.8490	-1.0 ± 2.8	0.0763	0.3150
Retrospective Memory Subscale	0.3 ± 2.4	0.6228	0.7 ± 3.2	0.3347	0.6995	-0.9 ± 3.0	0.1516	0.0915
Prospective Memory Subscale	0.3 ± 2.6	0.6432	-0.7 ± 2.9	0.2387	0.2527	-0.5 ± 2.8	0.3714	0.7500
Planning/Organization Subscale	-0.2 ± 1.8	0.6073	0.1 ± 3.9	0.8740	0.7031	-1.0 ± 2.1	0.0131	0.2053
PDQ Total Score	-0.4 ± 7.9	0.8325	-0.7 ± 12.2	0.8050	0.9412	-3.7 ± 8.8	0.0372	0.3209
PDQ 5 items Scale	0.1 ± 2.4	0.8420	0 ± 3.6	1.0000	0.9085	-0.6 ± 2.6	0.2756	0.5330
Mental Health Inventory **+**								
Anxiety Subscale	5.1 ± 17.6	0.2337	1.2 ± 19.4	0.7713	0.5017	9.1 ± 17.1	0.0086	0.1214
Depression Subscale	5.6 ± 18.5	0.2208	3.5 ± 18.2	0.3505	0.7267	7.5 ± 16.9	0.0261	0.4198
Behavior Control Subscale	3.6 ± 16.3	0.3590	1.5 ± 20.3	0.7280	0.7137	4.5 ± 13.7	0.0961	0.5292
Positive Affect Subscale	6.7 ± 15.6	0.0880	-3.1 ± 19.9	0.4491	0.0921	7.7 ± 14.6	0.0098	0.0287
Mental Health Inventory Total Score	4.7 ± 15.4	0.2144	0.5 ± 16.7	0.8931	0.4066	7.0 ± 13.6	0.0114	0.1270
Modified MOS Social Support Survey Score **+**	-0.6 ± 17.9	0.8968	-0.7 ± 21.1	0.8833	0.9877	2.3 ± 18.4	0.5108	0.5934
Expanded Disability Status Scale-	-0.8 ± 0.7	0.0002	-0.6 ± 1.2	0.0263	0.5462	-0.9 ± 0.8	< .0001	0.2025
Ashworth Scale score-	-1.3 ± 2.9	0.0833	-0.6 ± 1.4	0.0499	0.3640	-0.8 ± 2.5	0.0947	0.6682
Restless Legs Syndrome score-	0.05 ± 7.6	0.9761	0.2 ± 5.8	0.8622	0.9395	0.3 ± 6.1	0.7875	0.9511

**Table 8:** Baseline to 16 weeks. Comparisons between MBSI-I, eMBSI-I and SH cohorts on quality of life variables

**Variable**	MBSI-I (24)	MBSI-I (w/i grp.) *P* >|t|	SH (25)	SH (w/i grp.) *P* >|t|	MBSI-I vs. SH *P* >|t|	eMBSI-I (29)	eMBSI-I (w/i grp.) *P* >|t|	eMBSI-I vs. SH *P* >|t|
Quality of Life +								
Physical Function Scale	5.6 ± 11.8	0.0762	1.8 ± 22.6	0.7324	0.5124	4.2 ± 11.5	0.0735	0.6572
Role-Physical Scale	7.4 ± 21.2	0.1724	4.8 ± 30.2	0.4787	0.7670	7.7 ± 33.0	0.2457	0.7549
Bodily Pain Scale	7.9 ± 20.8	0.1345	-1.2 ± 13.0	0.6664	0.1243	4.8 ± 18.8	0.2031	0.2164
General Health Scale	1.2 ± 8.6	0.5726	-0.1 ± 9.9	0.9661	0.6745	0.5 ± 7.3	0.7384	0.8203
Vitality Scale	2.2 ± 19.9	0.6666	0.5 ± 18.4	0.9088	0.7833	4.4 ± 16.6	0.1872	0.4365
Social Functioning Scale	1.6 ± 13.6	0.6524	0.6 ± 23.01	0.9088	0.8685	4.3 ± 22.1	0.3269	0.5667
Role-Emotional Scale	-2.0 ± 30.0	0.7909	0.6 ± 41.6	0.2455	0.4745	6.2 ± 38.2	0.4083	0.1483
Mental Health Scale	1.3 ± 14.8	0.7402	-2.0 ± 16.7	0.5800	0.5385	4.0 ± 16.9	0.2491	0.2287
Physical Components Summary Scale	2.9 ± 4.8	0.0334	1.67 ± 7.2	0.3362	0.5762	1.4 ± 5.1	0.2042	0.8799
Mental Component Summary Scale	-0.2 ± 9.7	0.9230	-2.0 ± 9.7	0.3998	0.6143	2.8 ± 11.7	0.2683	0.1734
Modified Fatigue Impact Scale-								
Physical Subscale	-0.9 ± 4.7	0.4516	0.8 ± 4.9	0.4851	0.3031	-1.6 ± 4.8	0.0908	0.0975
Cognitive Subscale	-2.6 ± 5.0	0.0597	2.2 ± 6.2	0.1370	0.0191	-3.5 ± 5.1	0.0024	0.0018
Psychosocial Subscale	-0.2 ± 1.3	0.5930	-0.1 ± 1.7	0.7103	0.9366	-0.3 ± 1.3	0.2943	0.7725
Modified Fatigue Impact Scale Total Score	-3.4 ± 8.0	0.1200	3.0 ± 9.4	0.1696	0.0411	-5.4 ± 9.3	0.0097	0.0051
Modified fatigue 5 Items scale	-1.2 ± 2.5	0.0682	0.5 ± 2.8	0.4533	0.0655	-1.3 ± 2.7	0.0072	0.0200
MOS Pain Effects Scale-	-1.4 ± 5.3	0.2601	0.7 ± 4.2	0.4574	0.1642	-2.0 ± 5.0	0.0453	0.0509
Sexual Satisfaction Scale-	-0.7 ± 4.2	0.5959	-1.2 ± 3.6	0.1978	0.7298	-0.7 ± 3.7	0.4505	0.6812
Bladder Control Scale-	0.8 ± 3.0	0.3106	-0.7 ± 5.2	0.5155	0.2660	-0.1 ± 3.6	0.8320	0.6459
Bowel Control Scale-	1.1 ± 2.6	0.0886	-0.2 ± 5.0	0.8636	0.3079	0.3 ± 2.8	0.5452	0.6763
Impact of Visual Impairment Scale-	-0.3 ± 1.5	0.4507	0.2 ± 1.9	0.6487	0.4051	-0.3 ± 1.6	0.2937	0.3083
Self-Reported Cognitive Dysfunction-								
Attention/Concentration Subscale	-0.8 ± 3.0	0.2869	-0.3 ± 3.0	0.6188	0.6487	-0.9 ± 2.6	0.1021	0.5273
Retrospective Memory Subscale	0 ± 3.0	1.0000	0.4 ± 2.5	0.4997	0.6705	-0.8 ± 3.1	0.2095	0.1782
Prospective Memory Subscale	-1.1 ± 2.9	0.1435	-0.1 ± 2.1	0.8336	0.2510	-1.1 ± 2.6	0.0281	0.1463
Planning/Organization Subscale	-1.2 ± 2.3	0.0388	0.1 ± 3.0	0.8820	0.1379	-1.5 ± 2.1	0.0007	0.0331
PDQ Total Score	-3.1 ± 8.7	0.1530	-0.1 ± 7.5	0.9746	0.2835	-4.1 ± 7.8	0.0100	0.0923
PDQ 5 items Scale	-0.5 ± 2.2	0.3322	-0.5 ± 3.2	0.4291	0.9859	-0.3 ± 2.1	0.5313	0.7080
Mental Health Inventory								
Anxiety Subscale	4.2 ± 15.7	0.2706	2.9 ± 23.8	0.5728	0.8421	8.9 ± 15.4	0.0053	0.3169
Depression Subscale	0.8 ± 17.3	0.8401	-2.0 ± 19.4	0.6256	0.6264	4.1 ± 17.7	0.2313	0.2482
Behavior Control Subscale	0.8 ± 18.7	0.8525	-2.0 ± 23.9	0.6920	0.6792	1.8 ± 16.7	0.5770	0.5083
Positive Affect Subscale	3.9 ± 15.6	0.3045	-0.9 ± 21.6	0.8453	0.4350	5.4 ± 15.1	0.0722	0.2339
Mental Health Inventory Total Score	2.8 ± 14.7	0.4323	-0.5 ± 19.3	0.9129	0.5614	5.4 ± 13.8	0.0477	0.2166
Modified MOS Social Support Score	-3.1 ± 15.1	0.4014	2.5 ± 21.3	0.5880	0.3577	-1.6 ± 17.5	0.6306	0.4576
Expanded Disability Status Scale-	-0.3 ± 0.6	0.0419	-0.02 ± 0.8	0.9014	0.2118	-0.4 ± 0.9	0.0277	0.1345
Ashworth Scale score-	-0.6 ± 1.8	0.1860	-0.8 ± 2.1	0.0890	0.7431	-0.4 ± 1.7	0.1622	0.5275
Restless Legs Syndrome score-	-0.3 ± 8.7	0.8767	2.0 ± 7.8	0.2344	0.3713	1.3 ± 8.7	0.4381	0.7575

Within group MBSI-I cohorts, showed significant improvements in bowel function, the Expanded Disability Status Scale (EDSS) at 10 weeks and the Planning Organization Subscale of the Self-Reported Cognitive Dysfunction scale and EDSS at 16 weeks (Table 8). Similarly, SH scale showed improvement relative to baseline in the EDSS and Ashworth Scale score (spasticity) at 10 weeks.

### Adverse events

One participant reported experiencing severe pain and needed to take more pain medications than usual a few hours after attending mindfulness session number eight. Another participant reported pain, a popping sensation under her armpit and shortness of breath after a repositioning herself on a yoga mat while performing a body scan. Her symptoms apparently resolved after chiropractic treatment. Participants were instructed during sessions to avoid or modify any poses that exceeded their known physical limitations, and to ask their health care providers if they had any concerns.

## Discussion

This pilot study represents the first reported randomized controlled study of mindfulness training compared to an active comparator (SH) to treat chronic insomnia in persons with MS. Outcome measures utilized standard objective and subjective measures of sleep. Additional study design features included a 16 week follow up assessment to determine duration of effect, as well as an expanded eMBSI cohort that included the original MBSI-I cohort and ten individuals who completed the SH program and assessments and then crossed over to join the 10-week MBSI sessions and follow up evaluations. One prospective insomnia study of mindfulness in MS also used actigraphy and self-reported sleep measures to compare mindfulness delivered via videoconferencing vs. in person but used a nonrandomized study design by comparing mindfulness to a wait-list control group [52]. That study showed a significant improvement in SE in the videoconference group compare to the control group (*p* = 0.042).

SE in our study did not improve with MBSI-I compared with SH and, therefore, the primary hypothesis was not met. SE also did not improve within the MBSI-I groups nor the SH group. It should be noted that SE was higher than would have been expected for cohorts of persons with insomnia, approaching 90%, suggesting a ceiling effect. By contrast, SE in the Lorenz study was 56.7%. SE reported in other studies ranged from the low 70 s to mid-80 s, with SEs measured by actigraphy tending to be higher than those obtained from sleep diaries [26, 27, 30, 53, 54].

However, one component of SE did change significantly in the mindfulness groups in our study. We found that sleep time decreased significantly in the mindfulness groups, particularly in the post intervention period. Sleep time is a major component of both the numerator and denominator of SE, so overall sleep efficiency remained unchanged. Also of note, evening sleep time in our study averaged 6.8 h, well above that reported in the Lorenz study (5.2 h). This counterintuitive result in our study was also observed in a randomized study comparing a six-week mindfulness program to usual care in 79 breast cancer survivors with chronic insomnia [55]. That study reported longer wake periods in mindfulness participants compared to controls (61.3 vs. 51.4 min. respectively). The reasons for this are unclear, but this possible effect of mindfulness should be explored in future studies.

Our subjective secondary sleep measures confirmed that our study cohort had a moderate level of insomnia both on the PSQI and ISI, which suggest that our participants may have perceived less restful sleep because of overlapping problems with daytime fatigue. Both average scores for the PSQI and ISI, between 9 and 10 and 16 to 17, respectively, indicate moderate insomnia [41, 44].

Results of secondary sleep measures in the study showed significant benefits for the mindfulness cohorts, including the ISI and the component scores of the PSQL, e.g., sleep latency, day dysfunction due to sleepiness and overall sleep quality. Although these outcomes showed stronger within group effects than between mindfulness and SH cohorts.

This is consistent with observations found in similar studies of mindfulness treatment in nonMS populations [26, 30, 52, 54–58]. Theses stronger pre and post interventions within mindfulness cohorts than between comparator groups perhaps reflects small samples sizes [54, 56].

One interesting observation is that the ISI improved significantly in the SH cohort at the 16-week but not the 10-week assessment. This appears to be a relatively isolated assessment as none of the other sleep parameters showed improvement in the SH group. However, this does not rule out the continued benefits and possible practice effects of sleep hygiene principles, which makes the possibility of carryover benefits of SH participants in the eMBSI-I cohort relevant (discussed below).

While our data showed continued positive benefits to mindfulness on subjective sleep parameters extended to six weeks post intervention, one challenge is that the positive effects of mindfulness may not be long lived, or superior to SH over the longer term. A study of an online mindful meditation program in MS showed benefits in terms of quality of life, anxiety, depression and sleep post intervention but these effects were no longer significant at six months [59]. Another confounding effect is the time intervals measured by the PSQLI, which asks participants to rate their sleep quality over the previous four weeks. Therefore, for the MBSI-I and the eMSBI-I cohorts the 10-week assessment following the mindfulness course may reflect an interim treatment effect and the 16 week assessment may more truly reflect a post-treatment effect. However, The PSQI showed robust effects over both time periods for the MBSI-I groups. This would not apply to the SH cohort since there was only a single, much earlier intervention. Future study designs may address these issues.

In order to increase the power of the study, given the low numbers of participants, the MBSI-I was expanded to include participants who were originally part of the SH cohort and this eMBSI-I cohort generally showed more robust benefit in some sleep parameters than the smaller MBSI-I cohort. This could reflect greater statistical power to detect differences or carryover effects from the combined treatments. To adequately assess carryover effects in the study design, we would have had to control for this using a parallel extended design for both groups, which would have been impractical.

Other outcomes indicated significant benefits for MBSI-I relative to SH in self-report quality of life measures, similar to what has been reported in other studies [31–33, 56, 59–62]. This confirms data showing that MBSR practice results in improvement in several domains that affect MS participants’ quality of life, particularly, but not exclusively, in the domains of cognitive functioning, fatigue and mental health. While it is possible that improvements in these domains might result in improvement in sleep it may also be inferred that improvement in sleep through MBSR intervention programs might lead to better functioning in those domains.

### Limitations

The study is limited by the power to detect differences between MBSI-I and SH due to the low number of participants and the higher number of dropouts in the MBSI-I groups. This was partly compensated by the crossover of 10 SH participants into the extended MBSI-I cohort. MBSR programs require significant commitment of time and energy. However, this introduces possible carryover effects from the combined treatment. Perhaps, incorporating make up mindfulness classes into the protocol would help retain participants. There may also be inherent limitations with in-person mindfulness training for PWMS who have significant physical impairments and low energy reserves.

The study was not designed to differentiate the effects of mindfulness on PWMS by subtype of MS, e.g., relapsing remitting or progressive forms, or disease severity. We did not collect data on MS subtype, and would not have had the power to analyze outcomes based on this parameter. It can be inferred, based on the relatively low EDSS scores, that the participants had relapsing remitting multiple sclerosis (RRMS). Given that this is a preliminary study, we did not have the power to analyze outcomes based on MS subtypes or impairment. However, future studies might collect this data for analysis.

Another set of issues are raised by the use of consumer wrist band actigraphy to measure sleep. These devices are not designed for clinical research studies. While there are benefits of using wrist actigraphy, there also exists potential errors in validity, accuracy and reliability compared with PSG, considered the gold standard for sleep assessment [44, 45]. Furthermore, the algorithms used by various consumer actigraphs are proprietary and raw data is not provided [45]. Our data might have been strengthened by the use of sleep diaries as well as an accurate assessment of sleep stages. As devices become more accurate, perhaps more precise sleep data and better interpretation of the data will strengthen future studies.

A final challenge in our study was gaps in data collection from some participants as not all wore their Fitbits™ Charge 2 devices consistently or had technical issues uploading their data. Fortunately, this only required minor adjustments to the time intervals used in the data analysis as described in the Methods Section.

## Conclusion

Despite these limitations, findings from the current study suggest that mindfulness meditation provides some potential benefits in subjective measures of sleep and quality of life. This supports the need for larger studies to fully test potential benefit in sleep outcomes.

## Data Availability

The datasets used and/or analyzed during the current study are available from the corresponding author on reasonable request.
